# Primary Dedifferentiated Liposarcoma of the Duodenum: A Report of a Rare Case With a Review of the Literature

**DOI:** 10.7759/cureus.101948

**Published:** 2026-01-20

**Authors:** Kayra Cangoz, Muhammed Bahadir Avci, Merve Meryem Kiran, Firathan Sarialtin

**Affiliations:** 1 Department of General Surgery, Akdagmadeni Sehit Sinan Babacan State Hospital, Yozgat, TUR; 2 Department of General Surgery, Kahramankazan State Hospital, Ankara, TUR; 3 Department of Pathology, Ankara Bilkent City Hospital, Ankara, TUR; 4 Department of Radiology, Ankara Bilkent City Hospital, Ankara, TUR

**Keywords:** case report, dedifferentiated liposarcoma, duodenum, gastrointestinal tumor, surgery

## Abstract

Primary and metastatic tumors of the duodenum are rare, and primary duodenal dedifferentiated liposarcomas (DDLPS) of this region are exceptionally uncommon. DDLPS is an aggressive malignancy characterized by a high risk of recurrence and metastasis. This report describes the case of a 43-year-old woman who presented with a 20-day history of isolated abdominal pain. CT images demonstrated a 68 x 120 mm lesion involving the distal portions of the duodenum. Intraoperatively, the lesion was found to be confined to the third and fourth portions, and a segmental duodenal resection with duodenojejunostomy using a circular stapler was performed. During the postoperative period, the patient experienced transient cholestatic findings likely secondary to papillary obstruction. After clinical improvement, she was lost to follow-up for seven months during doxorubicin therapy and later re-presented with extensive metastatic disease, ultimately resulting in death. Given the extremely limited number of similar cases, no standardized treatment strategy exists. This case highlights the importance of considering DDLPS in the differential diagnosis of duodenal masses, even in patients presenting with nonspecific symptoms, and provides additional insight that may guide clinical decision-making in future cases.

## Introduction

Liposarcomas are among the most common malignant soft tissue tumors, and they predominantly occur in the retroperitoneum, extremities, and deep soft tissue of the trunk [1,2]. According to the 2020 World Health Organization Classification of Soft Tissue Tumors, malignant adipocytic tumors are classified into well-differentiated liposarcoma, dedifferentiated liposarcoma (DDLPS), myxoid liposarcoma, pleomorphic liposarcoma, and myxoid pleomorphic liposarcoma [3]. Primary and metastatic tumors of the duodenum are very rare [4]. Primary duodenal DDLPS tumors are extremely rare, aggressive tumors characterized by a high risk of recurrence and distant metastasis [5-8].

The standard treatment for DDLPS is wide-margin surgical resection similar to other sarcomas [7]. Surgical treatment of duodenal tumors is extremely important since the duodenum is located in the center of major structures and involves major operations such as the Whipple procedure [4]. The prognosis and treatment protocol of DDLPS are unclear due to the low number of cases documented in the literature. To the best of our knowledge, there are only five previously reported cases of DDLPS in the English literature [2,6-9]. The present report describes the case of a patient presenting with abdominal pain who was diagnosed with a duodenal DDLPS, together with the treatment strategy applied. We are reporting this case to add another documented example of primary DDLPS to the literature, which is an exceptionally uncommon entity. We also aim to share some diagnostic and perioperative insights that may help clinicians in the decision-making process when encountering similar cases.

## Case presentation

**Figure 1:**
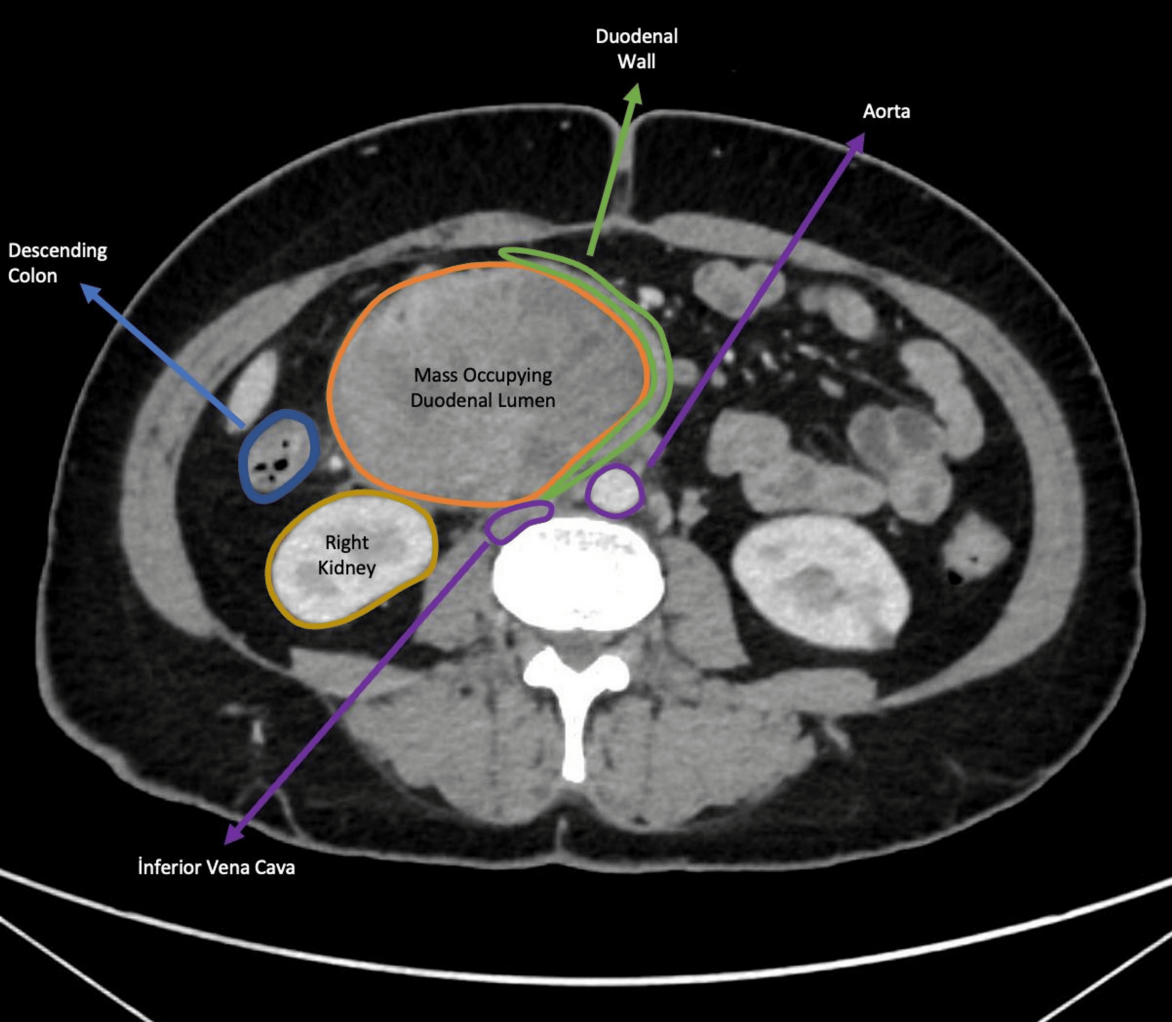
Post-contrast axial CT image demonstrating an intraluminal duodenal mass. A heterogeneously enhancing solid mass is seen within the duodenal lumen. No component extends beyond the lumen.

**Figure 2:**
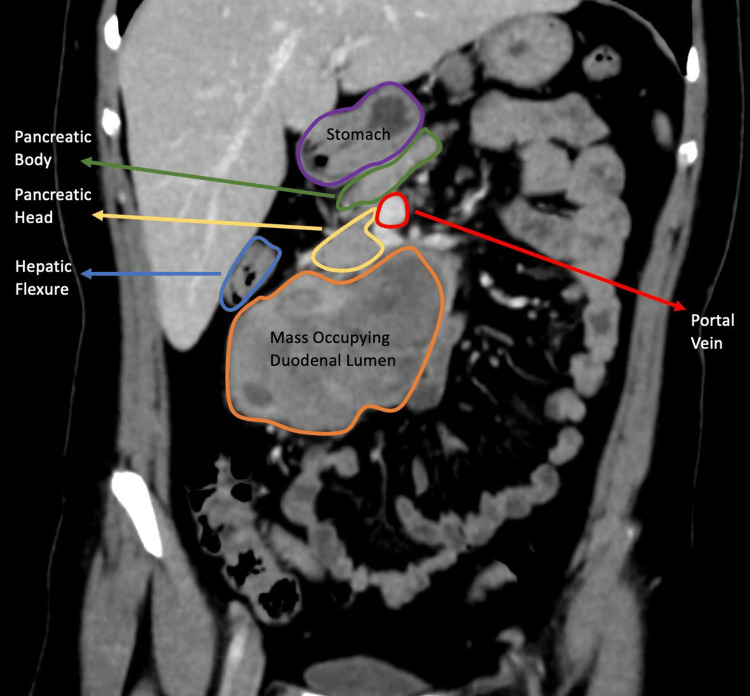
Contrast-enhanced coronal CT image of an intraluminal duodenal mass A large heterogeneously enhancing intraluminal duodenal mass is seen. Distally, the lesion extends along the lumen and forms an acute angle with the duodenal wall.

**Figure 3:**
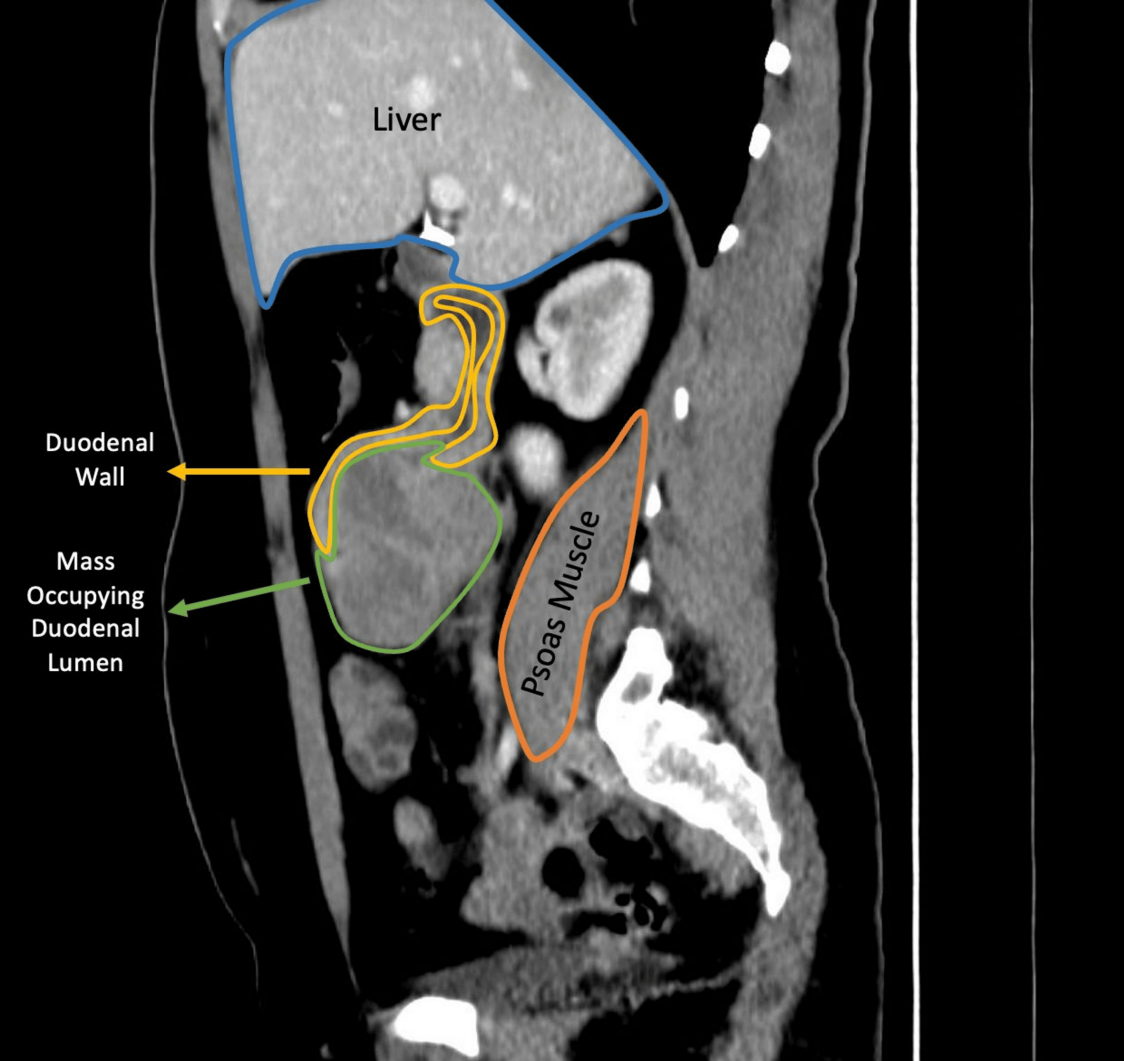
Contrast-enhanced sagittal CT image of a polypoid intraluminal duodenal mass A large heterogeneously enhancing intraluminal duodenal mass is seen. The lesion projects into the lumen in a polypoid fashion from the posterior wall, and its proximal margin forms an acute angulation with the duodenal wall.

A 43-year-old female patient presented to the emergency department with a 20-day history of abdominal pain. Her general condition was good, and her vital signs were stable. CT imaging revealed a heterogeneous soft tissue mass measuring 68 x 120 mm in the distal second and third parts of the duodenum. Duodenal luminal dilatation was observed at the level of the lesion, and multiple enlarged lymph nodes, the largest being 12 mm in diameter, were detected near the superior mesenteric artery (Figures 1-3). Upper endoscopy revealed a 6-cm lesion in the second part of the duodenum that prevented the passage of the endoscope. Biopsies were taken, and the result was reported as a malignant mesenchymal tumor consistent with DDLPS.

After the biopsy, further surgical intervention was planned. During the operation, it was observed that the mass did not extend to the second portion of the duodenum and was confined to the third and fourth parts. Therefore, a Whipple procedure was not performed. In order to preserve the pancreas and the ampulla of Vater, the third and fourth parts of the duodenum were resected, and a duodenojejunostomy anastomosis was created using a circular stapler.

**Figure 4:**
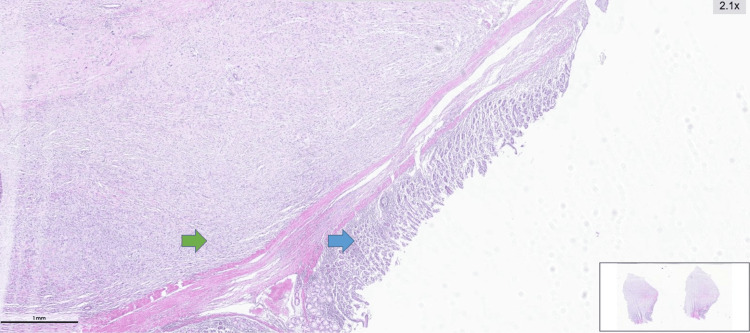
Histopathological appearance of the dedifferentiated liposarcoma showing preserved surface epithelium and an underlying spindle cell neoplastic proliferation. Blue arrow: Preserved surface epithelium. Green arrow: A well-circumscribed neoplastic proliferation composed of spindle cells, clearly demarcated from the epithelium.

**Figure 5:**
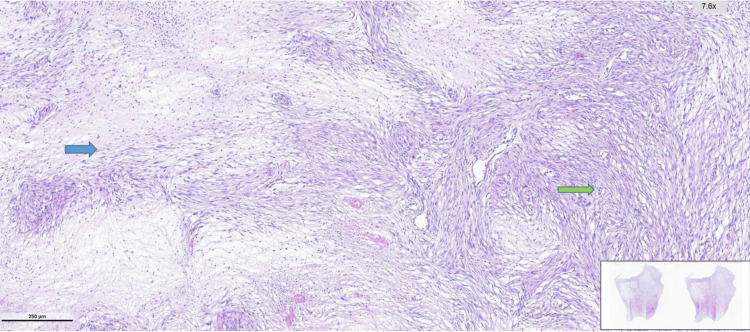
Hematoxylin and eosin–stained sections showing spindle cell neoplastic proliferation with alternating hypocellular and hypercellular areas Blue Arrow: Hypocellular area. Green Arrow: Hypercellular area

**Figure 6:**
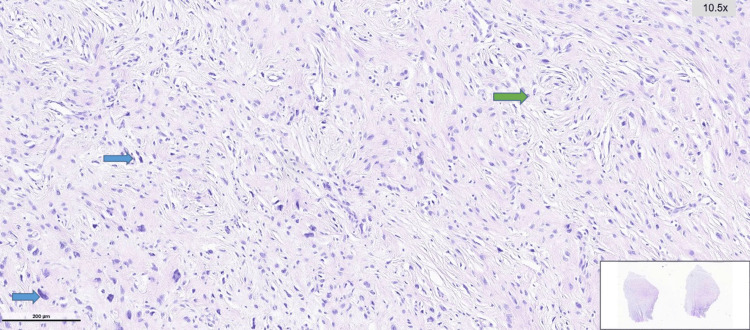
Hematoxylin and eosin–stained section showing spindle cell neoplastic proliferation with focal whorled architecture and short fascicular arrangements Blue Arrow: Areas with hyperchromatic and pleomorphic tumor cells. Green Arrow: Focal storiform growth pattern

**Figure 7:**
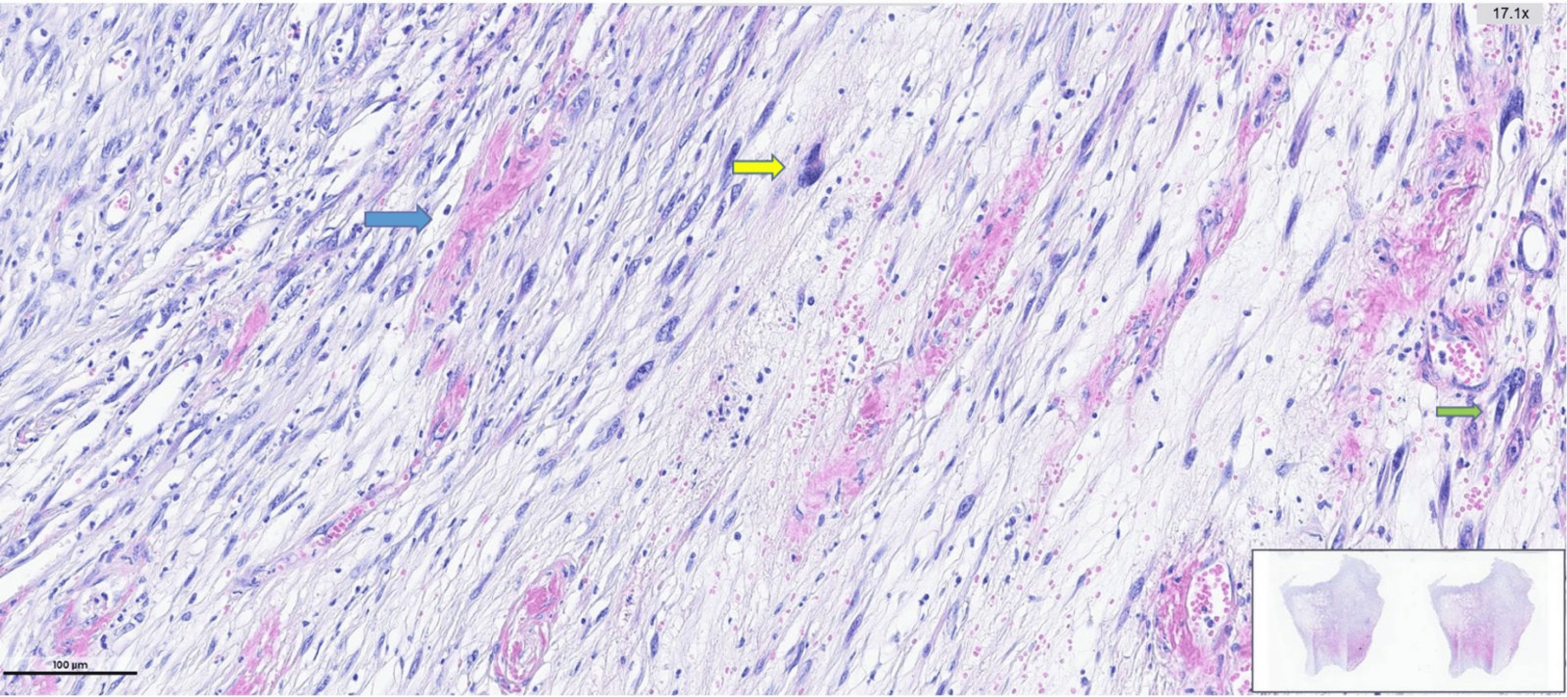
Hematoxylin and eosin–stained section showing spindle cell neoplastic proliferation with prominent vascularity Spindle cell neoplastic proliferation is with alternating hypocellular and hypercellular areas. Areas with prominent vascularity composed of spindle cells exhibiting hyperchromatic and pleomorphic nuclei are observed. Blue Arrow: Dilated, congested vascular structures. Yellow and Green Arrows: Hyperchromatic, pleomorphic tumor cells.

**Figure 8:**
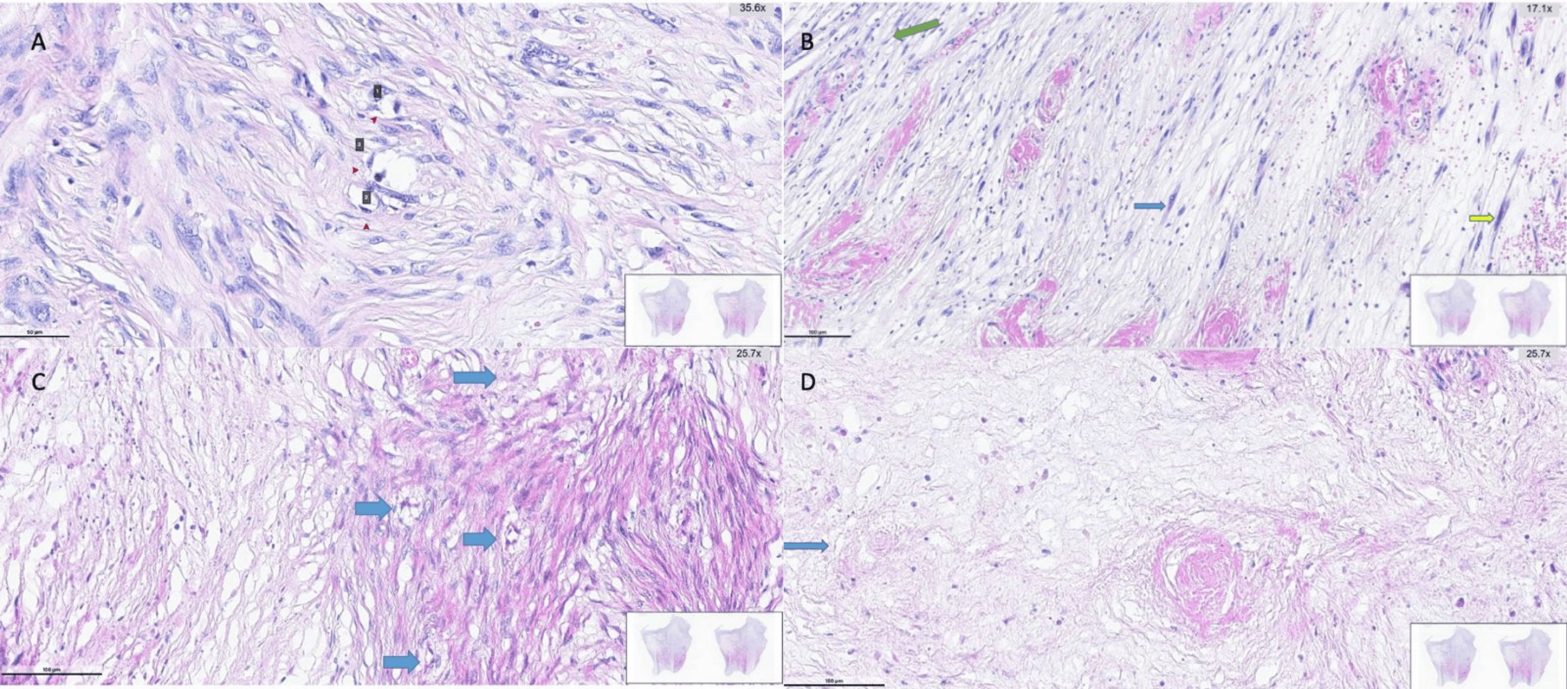
Histopathological features of dedifferentiated liposarcoma on hematoxylin and eosin staining (A) Arrows indicate lipoblasts; (B) Blue and yellow arrows indicate pleomorphic spindle-to-oval tumor cells; green arrow indicates inflammatory cells; (C) Blue arrows indicate lipoblasts characterized by vacuolated cytoplasm and centrally located nucleoli; (D) Necrotic area within the tumor.

Final pathology revealed atypical cells with increased MDM2 signal activity, consistent with DDLPS. Tumor cells were positive for Vimentin. The tumor cells were negative for C-kit, Desmin, SMA, S-100, Pancytokeratin, DOG1, Factor XIIIa, MUC4, ALK, SOX10, Melan-A, and HMB-45. The closest surgical margin measured 3 cm, and the tumor dimensions were 12 x 7 x 5 cm. The tumor invaded the muscularis propria, submucosa, and, focally, the mucosa. No tumor involvement was identified at the surgical margins (Figures 4-6). Cellular atypia was evident (Figure 7). Necrosis and atypical mitotic figures were identified (Figure 8).

Examination of 14 lymph nodes revealed no metastasis. Based on the American Joint Committee on Cancer (AJCC) soft tissue sarcoma staging system [10], the specimen corresponded to pT3N0M0. No vascular invasion was observed. Cellular atypia was evident. Necrosis and atypical mitotic figures were identified. Ki-67 proliferation index was 28%. Fluorescence in situ hybridization (FISH) performed using the Kreatech™ MDM2 (12q15)/SE 12 (Leica Biosystems Nussloch GmbH, Nussloch, Germany) set on a ThermoBrite® Elite system (Leica Biosystems Nussloch GmbH) demonstrated increased MDM2 signal activity in atypical tumor cells, confirming gene amplification. On postoperative day 8, the patient was discharged without complications.

On postoperative day 13, she presented to the emergency department again with abdominal pain. Laboratory tests showed elevated bilirubin levels (total bilirubin: 6.4 mg/dL and direct bilirubin: 5.0 mg/dL; adult female reference ranges: total 0.3-1.2 mg/dL, direct < 0.0.3 mg/dL), along with increased amylase, lipase, liver function tests, and cholestatic markers. Abdominal CT demonstrated increased pancreatic duct caliber, and hepatic ultrasonography revealed a proximal common bile duct diameter of 15 mm. This presentation was considered consistent with transient papillary obstruction due to inflammatory edema of the residual duodenal segment. She was admitted to the hospital and evaluated by the interventional radiology department. Due to no contrast passage from papilla to duodenum and the presence of intrahepatic and extrahepatic ductal dilatation, percutaneous transhepatic cholangiography (PTC) and external drainage were performed.

On postoperative day 25, she developed nausea and vomiting. She underwent upper gastrointestinal (GI) endoscopy, which showed no pathology at the anastomosis. Subsequently, endoscopic retrograde cholangiopancreatography (ERCP) was performed. Blue dye injected through the PTC catheter was observed entering the duodenum during the procedure; however, the papilla could not be visualized. By the second postoperative month, her bilirubin levels had normalized, but she required a secondary operation. Due to increased intra-abdominal inflammation, hepaticojejunostomy (HJ) could not be performed, and a gastrojejunostomy (GJ) was created. 

During her admission, her general status worsened in the third postoperative month, and she was transferred to the intensive care unit (ICU). During her ICU stay, she experienced seizures, which were evaluated by the neurology department and diagnosed as neuropathy of chronic disease. After a 10-day ICU admission, she was transferred back to the ward, and her general condition improved. Following normalization of her blood tests and clinical stabilization, her external drain was removed, and she was discharged.

**Figure 9:**
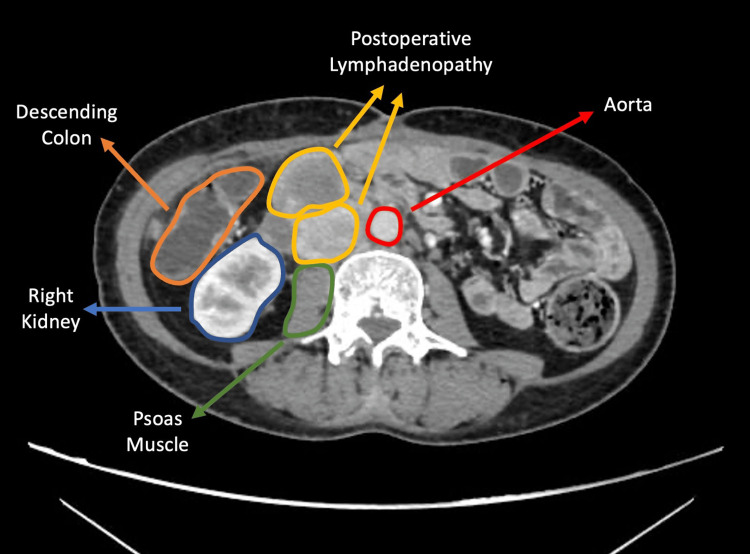
Contrast-enhanced axial CT image showing retroperitoneal lymphadenopathy Lymphadenopathies are seen anterior to the psoas muscle at the level of the renal hilum

Her prolonged postoperative hospitalization and subsequent development of significant cachexia after discharge resulted in poor functional status, limiting her eligibility for immediate adjuvant therapy. In the fifth postoperative month, she presented to the oncology department, and her follow-up CT imaging revealed three lymph nodes anterior to the inferior vena cava (IVC) at the level of the left renal hilum, the largest measuring 35 × 27 mm, exerting pressure on the IVC (Figure 9). Following multidisciplinary tumor board evaluation, single-agent doxorubicin therapy was recommended while immediate radiotherapy was not planned, considering the patient’s clinical condition and tumor-related features.

**Figure 10:**
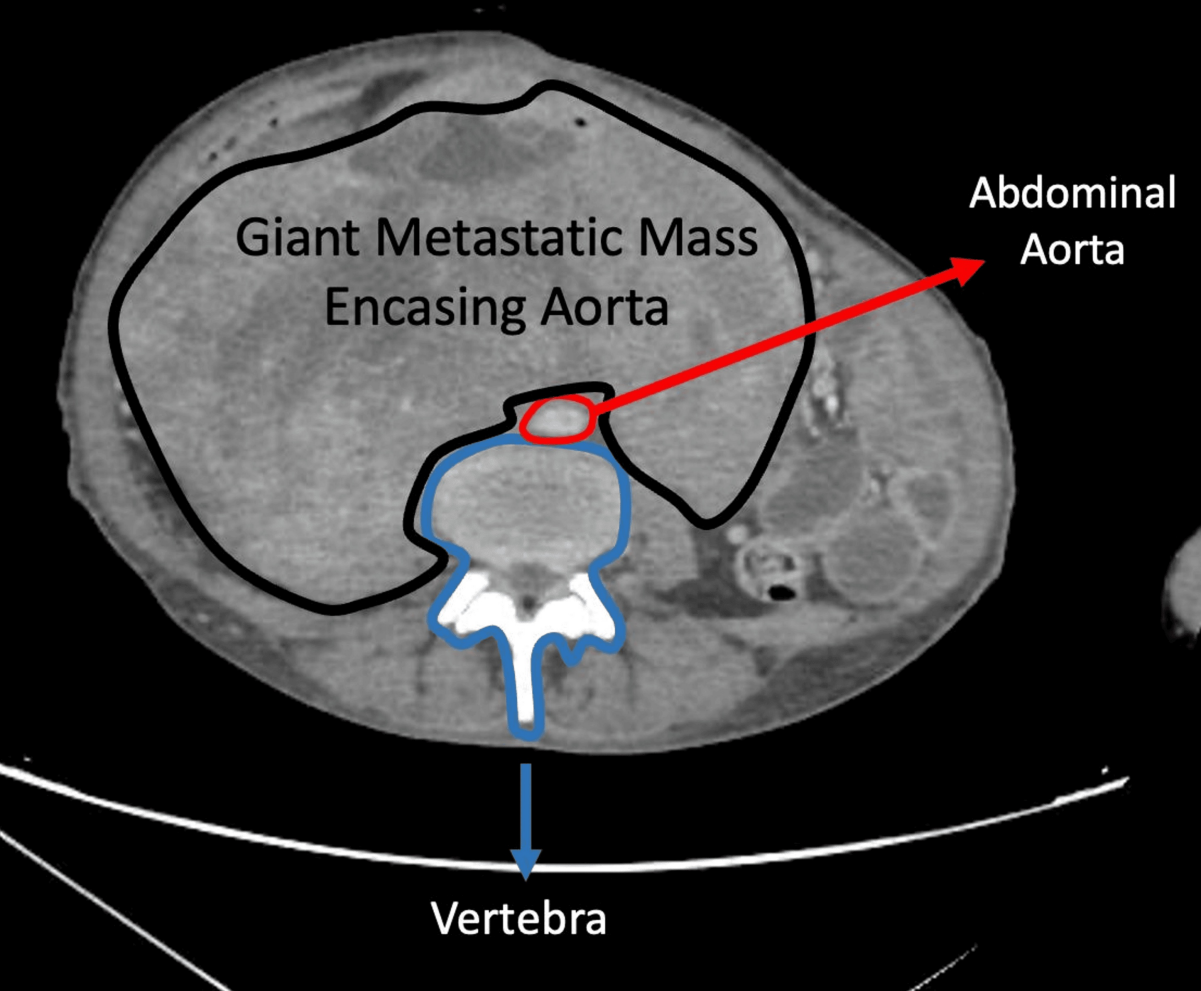
Contrast-enhanced axial CT image demonstrating a large retroperitoneal mass Post-contrast axial image showing a large mass encasing the aorta, extending into the paravertebral region, filling the right hemiabdomen, and displacing the small-bowel loops to the left

**Figure 11:**
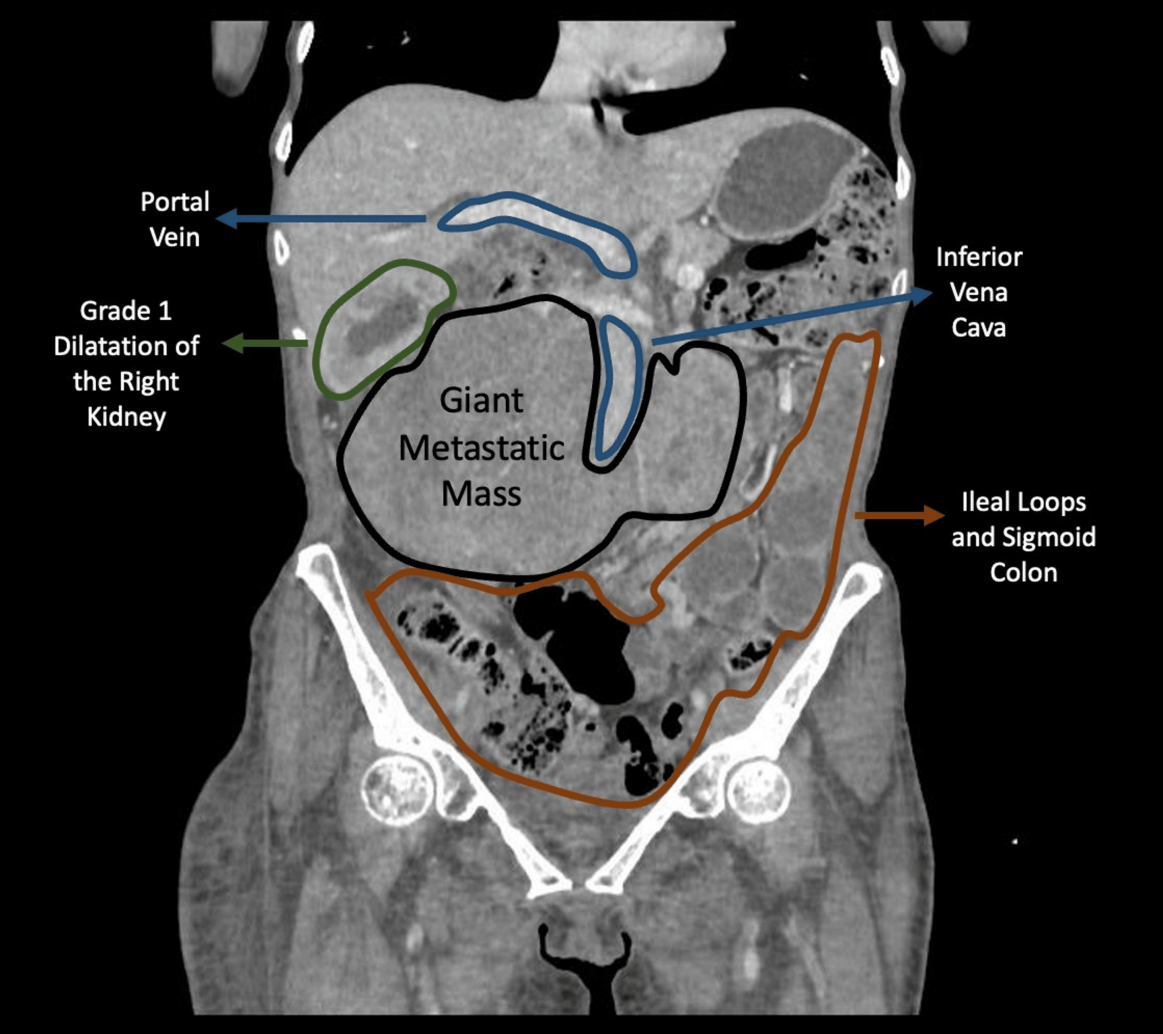
Contrast-enhanced coronal CT image demonstrating a large retroperitoneal mass Post-contrast coronal image demonstrating an enlarged mass encasing the aorta and dispşacing the right kidney superiorly

Shortly thereafter, the patient relocated to another city to continue treatment, interrupting her planned care at our center. She was subsequently lost to follow-up until 12 months postoperatively. Her CT imaging at that time, when she presented with bilateral grade-3 edema, revealed a 22 mm metastatic lesion in liver; a 20 x 15 x 20 cm metastatic lesion extending from the duodenum to the IVC and encasing the aorta 270 degrees (Figures 10, 11); a 21 mm metastatic nodule in right kidney invading the right renal artery and vein; and dilatation of the portal and splenic veins. Following these findings, she was referred to palliative care and subsequently died.

## Discussion

Primary liposarcomas located in the GI tract are extremely rare [2]. Furthermore, primary DDLPS is considered the rarest type of GI liposarcomas [8]. To the best of our knowledge, only five cases of primary duodenal DDLPS have been documented in the English literature [2,6-9]. The present case represents the sixth reported case, and it differs from previously published cases by its surgical management and postoperative course.

Published data indicate that adult soft tissue sarcomas often arise without well-established predisposing risk factors [11]. Our patient similarly had no identifiable environmental, hereditary, or iatrogenic risk factors. Furthermore, DDLPS can present with nonspecific symptoms, and GI stromal tumors (GIST), leiomyosarcoma (LMS), malignant melanoma, and sarcomatoid carcinoma should be considered in the differential diagnosis [2,6]. GISTs typically show expression of CD117, DOG1, and CD34, whereas sarcomatoid carcinomas can be identified by cytokeratin positivity due to their epithelial differentiation. LMS is usually positive for at least one myogenic marker, such as desmin or SMA [2]. Tumors with melanocytic differentiation commonly express S100, SOX10, HMB-45, and Melan-A [3]. In our case, negativity for CD117, DOG1, desmin, SMA, S-100, pancytokeratin, SOX10, Melan-A, and HMB-45, together with increased MDM2 signal activity, supported a definitive diagnosis of DDLPS, which is genomically characterized by amplification of the 12q13-15 region, particularly involving MDM2 and CDK4 [2]. 

Okabayashi et al. reported the first documented case of primary duodenal DDLPS in the literature. They described a 55-year-old patient with a 9.5 cm lesion located in the second portion of the duodenum, causing dilatation of the common bile duct, and showing invasion into the transverse colon [9]. Due to active hemorrhage, an emergency pylorus-preserving pancreaticoduodenectomy with partial resection of the transverse colon was performed. After a 10-month follow-up period, no recurrence was observed. They noted that endoscopic control of bleeding was difficult, arterial embolization around the duodenum could lead to ischemic necrosis, and catheter-based interventions would be insufficient due to complex arterial anastomoses in this region. Therefore, they concluded that their surgical approach represented the most appropriate technique for hemorrhagic primary duodenal liposarcoma [9].

Whitham et al. reported a case of a 59-year-old female patient who presented to the emergency department with fatigue, shortness of breath, and a hemoglobin level of 6.9 g/dL [8]. Upper endoscopy, performed due to suspicion of GI bleeding, revealed an ulcerated submucosal lesion on the posterior wall of the duodenal bulb. CT imaging confirmed a 5.2 x 4.9 x 4.8 cm heterogeneous mass. During surgery, pancreaticoduodenectomy was avoided since the lesion was confined to the first portion of the duodenum. Instead, they performed segmental duodenal resection and distal gastrectomy with Roux-en-Y reconstruction. The patient experienced no recurrence or distant metastasis during 16 months of postoperative follow-up. The authors emphasized that complete surgical resection remains the gold standard treatment for soft tissue sarcomas and offers the only chance for cure [8].

Kim et al. presented a 64-year-old male patient with a seven-day history of abdominal pain [2]. CT imaging revealed a 3 cm heterogeneous mass in the pancreaticoduodenal groove, causing obstruction of the second portion of the duodenum. Intraoperative findings demonstrated invasion into the pancreas, hepatic flexure, and adjacent major vessels; therefore, the patient underwent pylorus-preserving pancreaticoduodenectomy with en bloc right hemicolectomy, segmental resection of the superior mesenteric vein (SMV), and wedge resection of the IVC. Although the mass was resected, complete removal was not possible because of disseminated disease. The postoperative course was complicated by HJ leakage, peritonitis, sepsis, and multiple reoperations, and the patient died on postoperative day 60 due to disseminated intravascular coagulation. They emphasized that DDLPS of the small intestine may present with nonspecific symptoms and should be considered in the differential diagnosis of duodenal masses [2].

Alvi et al. reported a 66-year-old female patient who presented to the emergency department with syncope and melena with a hemoglobin level of 5.2g/dL [6]. CT imaging revealed a 6.5 x 4.7 cm lobulated soft-tissue mass arising from the superior wall of the duodenum, along with a 4 x 3 cm hepatic lesion. Endoscopy demonstrated a duodenal mass with active bleeding and gastric outlet obstruction. Arterial embolization was performed to control the bleeding, and GJ was subsequently created via laparotomy to address the obstruction. They emphasized that surgical resection is the first-line treatment for soft-tissue sarcomas and that achieving negative surgical margins improves overall survival. They also stated that radiotherapy and doxorubicin-based chemotherapy may provide additional benefit, although treatment should be individualized on a case-by-case basis, given the risk of radiation-related toxicity to intra-abdominal organs [6]. In our patient, immediate postoperative radiotherapy was not administered due to the patient’s postoperative clinical condition and tumor-related features, as concluded by the multidisciplinary tumor board.

Finally, Noguchi et al. described a 51-year-old woman who presented to the emergency department with syncope and a hemoglobin level of 4.5 g/dL [7]. CT imaging demonstrated a 7 cm mass centered in the horizontal portion of the duodenum, accompanied by multiple mesenteric and pancreatic lesions. Endoscopy revealed a solid tumor with active bleeding. A non-surgical approach was adopted, and a GJ was performed to relieve newly developed GI obstruction. The patient subsequently received systemic chemotherapy with eribulin and radiotherapy. However, she died nine months after the initial presentation. The authors noted that this was the first reported duodenal DDLPS managed with a combination of radiotherapy and chemotherapy. They also emphasized that, although surgical resection remains the most effective curative, unresectability may occur in some cases. Duodenal liposarcomas appear to present more frequently with bleeding compared to liposarcomas in other segments of the small intestine, which more commonly cause obstruction [7].

In our case, the patient was the youngest among the previously reported cases and had the largest primary lesion (Table 1). As noted by Noguchi et al. [7], larger liposarcomas may be associated with a higher likelihood of recurrence and mortality, although available data remain limited. This observation may partially explain the aggressive metastatic progression seen in our patient following surgery. Because the tumor did not extend to the second portion of the duodenum, we performed a segmental resection with duodenojejunostomy using a circular stapler. Atie et al. stated that postoperative edema can occur in anastomosis following gastrointestinal surgery and can cause partial obstruction [12]. Additionally, Takada et al. reported that interventions involving the duodenum can lead to papillary edema and subsequent bile-flow obstruction [13]. Considering this mechanism, postoperative edema in the remaining duodenal segment may have transiently obstructed the ampulla of Vater, contributing to the cholestatic findings observed during follow-up. 

**Table 1:** Primary duodenal DDLPS cases reported in the literature CBD: common bile duct, SOB: shortness of breath, SMV: superior mesenteric artery, IVC: inferior vena cava, DIC: disseminated intravascular coagulopathy, GJ: gastrojejunostomy, GOO: gastric outlet obstruction; M: male; F: female

Author(s), Year	Age (years)/ Sex	Presentation	Tumor Size & Location	Surgery / Treatment	Outcome
Okabayashi et al., 2013 [9]	55/M	Melena	9.5 cm – 2^nd^ portion of duodenum; invasion to transverse colon; CBD dilatation	Emergency pylorus-preserving pancreaticoduodenectomy + Partial transverse colon resection	No recurrence at 10 months
Whitham et al., 2020 [8]	59/F	Fatigue, SOB, Anemia (Hb: 6.9)	5.2 x 4.9 x 4.8 cm – duodenal bulb (1^st^portion), ulcerated	Segmental duodenal resection + Distal gastrectomy (Roux-en-Y)	No recurrence or metastasis at 16 months
Kim et al., 2022 [2]	64/M	7-day abdominal pain, weight loss	3 cm – pancreaticoduodenal groove causing obstruction of 2^nd^portion, invasion to the pancreas, hepatic flexure and adjacent major vessels	Pylorus-preserving pancreaticoduodenectomy + En bloc right hemicolectomy + SMV segmental resection + IVC wedge resection	Complicated postoperatively, died on postoperative day 60 due to DIC
Alvi and Shankar, 2023 [6]	66/F	Syncope, Melena (Hb: 5.2)	6.5 x 4.7 cm – superior duodenal wall; concurrent liver lesion	Arterial embolization for bleeding + GJ for GOO	Long-term outcome not reported
Noguchi et al., 2025 [7]	51/F	Syncope (Hb: 4.5)	7 cm – horizontal part; multiple pancreatic and mesenteric lesions	Unresectable – RT + Eribulin chemotherapy + GJ	Died 9 months after diagnosis
Present Case (Cangoz et al.), 2026	43/F	20-day abdominal pain	68 x 120 mm – distal 2^nd^ and 3^rd^ portions of duodenum (intraoperative 3^rd^ and 4^th^ portions only)	Segmental duodenal resection (3^rd^ and 4^th^portions) + duodenojejunostomy using circular stapler	Transient papillary obstruction; Lost to follow-up; Massive metastatic relapse; Died at 12 months

Although the surgical margins were negative (R0 resection), the locoregional lymph-node recurrences identified at the fifth postoperative month, combined with the patient's subsequent seven-month loss to follow-up, likely allowed the disease, characterized by the inherently aggressive biology of DDLPS, to progress unchecked. By the time of re-presentation at the 12th month, the massive retroperitoneal mass encasing 270 degrees of the aorta and compressing the IVC represented not a continuation of the original lesion, but rather the result of this rapid and destructive recurrence. This case underscores the critical importance of uninterrupted oncologic surveillance and patient adherence following surgery for DDLPS.

## Conclusions

We reported a case of primary duodenal DDLPS, an exceedingly rare entity. This case highlights the importance of including DDLPS in the differential diagnosis of duodenal tumors, even in patients presenting with nonspecific symptoms such as isolated abdominal pain. The primary lesion was large, which was followed by rapid metastatic progression and clinical deterioration. Given the rarity, no standardized treatment approach for surgical strategy has been established for duodenal DDLPS. Therefore, we believe that this case adds valuable insight that may assist clinicians in the diagnostic evaluation and management of future patients.
